# Management of severe strongyloidiasis attended at reference centers in Spain

**DOI:** 10.1371/journal.pntd.0006272

**Published:** 2018-02-23

**Authors:** Angela Martinez-Perez, Silvia Roure Díez, Moncef Belhassen-Garcia, Diego Torrús-Tendero, Jose Luis Perez-Arellano, Teresa Cabezas, Cristina Soler, Marta Díaz-Menéndez, Miriam Navarro, Begoña Treviño, Fernando Salvador, the Soil-Transmitted Helminths’ Study Group of the Spanish Society of Tropical Medicine and International Health (SEMTSI)

**Affiliations:** 1 Consorcio de Atención Primaria en Salud Barcelona Esquerra, Barcelona, Spain; 2 Hospital Universitari Germans Trias i Pujol, Enfermedades Infecciosas, PROSICS Metropolitana Nord, Badalona, Spain; 3 Hospital Universitario de Salamanca, Servicio Medicina Interna, Sección Enfermedades Infecciosas, Centro de Investigación de Enfermedades Tropicales de la Universidad de Salamanca, CAUSA, IBSAL, CIETUS, Universidad de Salamanca, Salamanca, Spain; 4 Hospital General Universitario de Alicante, Enfermedades Infecciosas, Medicina Tropical, Alicante, Spain; 5 Complejo Hospitalario Universitario Insular Gran Canaria, CHUIMI, Unidad de Enfermedades Infecciosas y Medicina Tropical, Las Palmas de Gran Canaria, Spain; 6 Hospital de Poniente, Microbiology, Medicina Tropical, Almeria, Spain; 7 Hospital Santa Caterina, Enfermedades Infecciosas, Unitat de Salut Internacional i Medicina Tropical, Salt/Girona, Spain; 8 Hospital Universitario La Paz-Carlos III, Enfermedades Infecciosas, Unidad de Medicina Tropical y del Viajero, Madrid, Spain; 9 Spanish Society of Tropical Medicine and International Health SEMTSI, Madrid, Spain; 10 Vall de Hebrón University Hospital, Tropical Medicine Unit, Barcelona, Spain; 11 Infectious Diseases Department, Vall de Hebrón University Hospital, PROSICS, Barcelona, Spain; Sacro Cuore Hospital, ITALY

## Abstract

**Introduction:**

*Strongyloides stercoralis* is a globally distributed nematode that causes diverse clinical symptoms in humans. Spain, once considered an endemic country, has experienced a recent increase in imported cases. The introduction of serology helps diagnosis and is currently replacing microbiological techniques in some settings, but its sensitivity is variable and can be low in immunocompromised patients. Diagnosis can only be confirmed by identification of larvae. Often, this “gold standard” can only be achieved in severe cases, such as disseminated *S*.*stercoralis* infection, or *S*.*stercoralis* hyperinfection syndrome, where parasite load is high. In addition, these clinical presentations are not well-defined. Our aim is to describe severe cases of *S*.*stercoralis*, their epidemiological profile, and their clinical details.

**Methods:**

An observational retrospective study of disseminated *S*.*stercoralis* infection, or hyperinfection syndrome. Inclusion criteria: aged over 18, with a diagnosis of disseminated *S*.*stercoralis* infection, or hyperinfection syndrome, confirmed by visualization of larvae. Patients were identified through revision of clinical records for the period 2000–2015, in collaboration with eight reference centers throughout Spain.

**Results:**

From the period 2000–2015, eighteen cases were identified, 66.7% of which were male, with a median age of 40 (range 21–70). Most of them were foreigners (94.4%), mainly from Latin America (82.3%) or Western Africa (17.6%). Only one autochthonous case was identified, from 2006. Immunosuppressive conditions were present in fourteen (77%) patients, mainly due steroids use and to retroviral coinfections (four HIV, two HTLV). Transplant preceded the clinical presentation in four of them. Other comorbidities were coinfection with HBV, *Trypanosoma cruzi*, *Mycobacterium leprae* or *Aspergillus spp*. All presented with digestive disorders, with 55.6% also presenting malaise. 44.4% of cases had fever, 27.8% skin complaints, and 16.7% respiratory or neurological disorders. One patient presented anemia, and one other nephrotic syndrome. Diagnosis was confirmed by identification of larvae in fresh stool samples (n = 16; 88.9%), concentration techniques (n = 6; 33.3%), larval culture (n = 5; 29.4%), or digestive biopsies (n = 8; 44%). *S*.*stercoralis* forms were identified during necropsy in one case. In addition, ten (55%) had a positive serology. All the cases were treated with ivermectin, six (33%) also received albendazole and one case received thiabendazole followed by ivermectin. All needed inpatient management, involving a mean hospitalization stay of 25 days (range 1–164). Two cases received intensive care and eventually died.

**Conclusions:**

Only eighteen cases of disseminated *S*.*stercoralis* infection/hyperinfection syndrome were identified from the 15-year period, most of which were considered to have been imported cases. Among those, immunosuppression was frequent, and mortality due to *S*.*stercoralis* was lower than previously described.

## Author summary

*Strongyloides stercoralis* is a globally distributed worm. It has a free living cycle in wet moist soils, and an autoinfecting cycle affecting humans in their lungs, bowels and skin. Strongyloidiasis is the name of the infection caused by *S*.*stercoralis* and it can vary from an indolent state, with no symptoms at all, to a severe clinical condition if the adult worms reproduce and disseminate into the body tissues. This second clinical picture usually presents when the immune system of the host is altered for any reason, most likely other immunosuppressive infections or treatments. Severe clinical conditions are usually confirmed when an increased number of *S*.*stercoralis* larvae are found in body tissues, which leads to a broad spectrum of clinical symptoms that are usually fatal without treatment. These conditions are both uncommon and not well defined. We present the experience of eight referral centers in Spain regarding the management of severe strongyloidiasis. We found 18 cases diagnosed over 15 years, the second largest series outside endemic areas, and compiled their epidemiological, clinical and outcome data.

## Introduction

Strongyloidiasis is an infection caused by *Strongyloides stercoralis*. Other *Strongyloides spp*. include *S*. *fuelleborni*, which infects some non-human primates and may cause self-limited disease in humans. *S*.*stercoralis* is widely distributed, affecting up to 370 million people worldwide according to recent estimates [1, 2], and some experts claim that its prevalence is increasing around the globe [1, 3]. Spain had an estimated prevalence of 0.2% in some rural areas during the first half of the last century [4], especially where wet crops, such as rice, and animal tracks were common.

The epidemiology of the disease in Spain changed notably in the early 80s owing to economic growth, the abandonment of traditional farming techniques and the mechanization of agriculture, along with an improvement in sanitation networks in rural areas. Although, some reports suggest that such transmission may still be occurring, [5, 6], all these advances have led to autochthonous cases becoming rare [5].

Economic growth brings both population ageing and an increase in the availability of diagnosis and treatment for chronic conditions, leading to a higher rate of comorbidities and also immunosuppressions in which severe cases might appear [6]. Recently published data from our group have shown a tenfold increase in hospital admissions where *S*.*stercoralis* is involved over the last decade [7].

This parasite undergoes a fascinating life-cycle that alternates between a free-living cycle in moist soil and a parasitic cycle in the host. The latter may last for decades, since the worm can replicate in the host and cause repeated autoinfections [8]. Parasite transmission to humans occurs during its free-living cycle through direct skin contact with previously contaminated soil containing rhabditiform larvae, although transmission through organ transplant has also been described [9, 10]. Therefore, poor sanitary conditions facilitate parasite transmission.

Strongyloidiasis is an indolent infection in most cases, although it can lead to mild digestive, skin or pulmonary symptoms. In some cases, usually due to comorbidities such as immunosuppression, replication of larvae can increase and they can disseminate into other tissues causing acute severe conditions [9]. These life-threatening forms of the disease comprise hyperinfection syndrome and disseminated strongyloidiasis. Both clinical pictures display a variety of symptoms, often with significant digestive involvement, that can produce paralytic ileus. In addition, larvae migration to the lungs can lead to hemoptysis and respiratory distress, and invasion of the brain can present as meningoencephalitis. Translocation of bowel bacteria causing sepsis and meningitis is often seen along with these findings. In both presentations, eosinophilia is an unfrequent finding and mortality can be as high as 62.7% [9]. Unfortunately, confirmation of diagnosis relies on visualization of parasite forms, which is unlikely with low parasite loads. Nevertheless, hyperinfection and disseminated forms are, by definition, situations where parasite load is high, so diagnosis in these cases is often confirmed.

As a Neglected Tropical Disease, strongyloidiasis is often forgotten about even where it is most common, and more so where it was once prevalent, but is no longer. Some authors state that *S*.*stercoralis* hyperinfection syndrome is in fact an emerging disease, and point to a lack of awareness among health-care professionals in non-endemic areas [11]. In particular, the clinical presentation of disseminated strongyloidiasis, or hyperinfection syndrome, is poorly defined in the literature, often based solely on case reports [6, 9]. Our aim is to define these severe forms of the disease as seen in our context.

## Methods

This is an observational retrospective study of disseminated *S*.*stercoralis* infection, or hyperinfection syndrome, identified through revision of clinical records. Cases were tracked in collaboration with the Geohelminths Study Group of the Spanish Society of Tropical Medicine and International Health (SEMTSI). The period studied was defined as from 1 January 2000 through 31 December 2015.

Inclusion criteria: aged over 18, with a diagnosis of disseminated *S*.*stercoralis* infection, or hyperinfection syndrome, confirmed by biopsy, necropsy, or microbiological evidence of *S*.*stercoralis* adult forms, eggs or larvae. Hyperinfection was defined as the presence of signs or symptoms suggesting increased larvae migration and accelerated autoinfection, with microbiological evidence for it. Migration of larvae out of the skin, lungs or digestive tract was regarded as dissemination.

Exclusion criteria: a lack of a parasitological confirmation test, and/or simple classical forms of the disease.

Study variables included age, gender, country of birth, occupational history, whether the case was considered to be imported or autochthonous, duration of residence in Spain, clinical manifestations, diagnostic evidence, treatment received, and main outcomes observed.

Eosinophilia was defined as an eosinophil count in peripheral blood exceeding 4.5×10^8^/L (450/μL) or more than 5% of the circulating leukocytes, in accordance with the consensus of the Spanish Society of Tropical Medicine and International Health expert group [12]. Data were collected through an anonymized on-line questionnaire, completed by study collaborators. Qualitative variables are expressed in absolute and relative frequencies, while continuous variables are described in medians and absolute ranges.

### Ethics statement

This work was performed in accordance with the ethical standards laid down in the Declaration of Helsinki as revised in 2013. The study protocol was approved by the Ethical Review Board of Vall de Hebron University Hospital (Barcelona, Spain) with the assigned code PR_AG_03–2016. Since this was a retrospective observational study, our Institutional Review Board accepted to proceed to data compilation and analysis with no previous informed consent obtained from the participants. All clinical and epidemiological data were anonymized.

## Results

Of the ten reference centers which participated in the study, two found no cases of disseminated strongyloidiasis nor hyperinfection syndrome, while the eight remaining centers were able to recover cases from their clinical records for the period defined. From the years 2000–2015, eighteen cases were identified. The majority were male (n = 12, 66.7%), which implies a sex ratio of 2:1, and the median age was 40 (range 21–69). Most of them were foreigners (n = 17, 94.4%), mainly from Latin America (n = 14, 82.3%) or Western Africa (n = 3, 17.6%). Of these patients, fourteen had information about the length of their period of residence in Spain, the median period being 7.21 years (range 2 months-31 years), and in two cases, it was possible to identify a recent travel history involving a visit to friends and relatives in their native country, respectively six and eleven months before the onset of symptoms. Further epidemiological details are provided in Table 1. Only one autochthonous case was identified, that of a 40-year-old construction worker from the Canary Islands with no travel history outside of Spain. He reported having walked barefoot in mud in a known endemic area in mainland Spain, and later developed hyperinfection syndrome after induced immunosuppression for a renal transplant.

**Table 1:** Epidemiological details.

Case number	Age (in years) & Sex	Country of Origin	Time residing in Spain (in years)	Occupation
1	28F	Paraguay	6	Cleaning lady
2	69F	Colombia	7 (last visit 11 months ago)	Housekeeper
3	44M	Peru	14 (last visit 6 years ago)	Laborer
4	47M	Ecuador	5	Unknown
5	25M	Dominican Republic	2 months	Unknown
6	21F	Brazil	1	Unknown
7	40M	Bolivia	4	Farmer (Bolivia) Laborer (Spain)
8	43M	Colombia	Unknown	Unknown
9	32F	Bolivia	9 months	Unknown
10	49M	Honduras	2	Cattle breeder
11	40F	Cuba	10	Hairdresser
12	41F	Colombia	4	Laborer (Colombia) Cleaning lady (Spain)
13	40M	Spain	Not Applicable	Construction worker.
14	40M	Peru	Unknown	Shipper
15	37M	Gambia	Unknown	Farmer
16	34M	Guinea Bissau	5	Shipper
17	42M	Colombia	11	Construction worker
18	43M	Equatorial Guinea	31	Laborer

F: Female, M: Male

Most of the cases fulfilled the criteria for hyperinfection syndrome (n = 17, 94.4%), and only one case of dissemination was identified and confirmed by necropsy (case 3). This allowed for identification of *S*.*stercoralis* in skin, lungs, bowels, brain, kidneys and lymph nodes. The clinical details of each patient are specified in Table 2 and summarized below.

**Table 2:** Clinical details.

Case number	Comorbidities	Clinical Manifestations at diagnosis	Date of diagnosis & Methods used	Hospital stay	Strongyloidiasis Treatment	Concomitant Treatment	Outcome
1	HTLV-1 infection	Digestive General malaise No Eosinophilia	December 2014: *S*. *stercoralis* in duodenal biopsy Serology +	26 days	Ivermectin 200mcg/Kg/day for 7 days	None	Alive
2	Renal transplant Smoker, COPD	Digestive Respiratory General malaise Eosinophilia	July 2013: *S*.*stercoralis* in bronchial aspiration and fecal samples Serology +	10 days	Ivermectin 200mcg/Kg/day for 2 days	Inhaled corticosteroids Prednisolone 20 mg/day Mycophenolic Acid 500 mg/12H	Alive
3	Chronic myeloid leukemia Allogenic peripheral blood stem cell transplant	Digestive Respiratory Skin Neurological General malaise Eosinophilia present in historical records before admission	March 2014: *S*.*stercoralis* in bronchial aspiration Necropsy Serology not requested	164 days, in ICU	Ivermectin 200mcg/Kg/day + Albendazole 400mg/day for 2 days	Prednisolone 60 mg/day Tacrolimus 1.5mg/day	Dead
4	Unknown	Digestive Fever Eosinophilia	March 2010: *S*.*stercoralis* in upper endoscopic biopsies, peritoneal fluid and fecal samples Serology +	10 days	Ivermectin 200mcg/Kg for 7 days	None	Alive
5	HIV infection Cerebral toxoplasmosis	Digestive Fever General malaise No Eosinophilia	March 2012: *S*.*stercoralis* in duodenal biopsy and fecal samples Serology +	14 days	Ivermectin 200mcg/Kg for 2 days (repeated after one week)	Prednisone 30 mg/day	Alive
6	HBV chronic infection	Digestive Respiratory Skin Fever General malaise Eosinophilia	October 2007: *S*.*stercoralis* in fecal samples, digestive fluids and duodenal biopsy Serology not requested	2 days	Ivermectin 200 mcg/Kg single dose during hospital stay and a second and third course 2 and 3 weeks after discharge	None	Lost to follow up
7	Renal transplant due to IgA nephropathy with severe nephrosclerosis Chagas disease	Digestive Skin General malaise Eosinophilia	April 2008: *S*.*stercoralis* in digestive fluids and fecal samples Serology +	1 day	Ivermectin 200 mcg/Kg/day for 2 days and again after 2 weeks	Tacrolimus 6.5mg/day Prednisone 10 mg/day	Alive
8	HIV infection HBV chronic infection	Digestive Neurological (myelitis) Skin Fever General malaise Eosinophilia	August 2008: *S*.*stercoralis* larvae in fresh fecal samples Serology not requested	30 days	Ivermectin 200 mcg/Kg/day + Albendazole 400mg/day for 7 days	Lopinavir/Ritonavir 133/33 mg /12hours Emtricitabine /Tenofovir 200/245 mg day	Alive
9	HIV infection Cerebral Toxoplasmosis Chagas disease	Digestive Respiratory Skin Fever No Eosinophilia	January 2012: *S*.*stercoralis* larvae in fresh fecal samples and sputum Serology not requested	10 days	Albendazole 400mg/day for 14 days + Ivermectin 200 mcg/Kg/day for 7 days and every 6 months until CD4 count recovery	Dexamethasone 2 mg/day Sulfadiazine 1g/6 hours Pyrimethamine 50 mg/day Darunavir/Ritonavir 800/400 mg/day Emtricitabine /Tenofovir 200/245 mg day	Alive
10	Unknown	Digestive General malaise Eosinophilia	March 2008: *S*.*stercoralis* larvae and adult forms in fresh fecal samples and gastric, duodenal and colonic biopsies Serology +	20 days	Thiabendazole 1750mg/day for 3 days + Ivermectin 200 mcg/Kg single dose	Diclofenac 50 mg Paracetamol 1 g Gabapentine 300 mg	Alive
11	Sjögren syndrome Renal transplant due to tubular nephritis Previous episodes of acute meningitis and bacterial peritonitis	Digestive Skin Neurological Fever General malaise Eosinophilia	September 2004: *S*.*stercoralis* larvae in fresh fecal samples and sputum Serology not requested	11 days	Ivermectin 200 mcg/Kg/day for 10 days	Prednisone 30 mg/day	Alive
12	Invasive pulmonary aspergillosis 2 months before onset	Digestive Fever General malaise Eosinophilia	February 2012: *S*.*stercoralis* larvae in fresh fecal samples, gastric fluids and duodenal biopsies. Serology not requested	14 days	Ivemectin 200 mcg/Kg/day for 4 days	Voriconazole 200 mg/12 hours Prednisone 30 mg/day	Alive
13	Renal transplant subsequent to hypertensive renal failure	Digestive Fever General malaise Eosinophilia	December 2006: *S*.*stercoralis* larvae in fresh fecal samples Serology not requested	6 days	Ivermectin 200 mcg/Kg for 10 days	Prednisone 10 mg/day Tacrolimus 1 mg/12H Mycophenolic Acid 500 mg/12H	Alive
14	HTLV-1 infection	Digestive General malaise Anemia No Eosinophilia	December 2011: *S*.*stercoralis* larvae in fresh fecal samples Serology +	7 days	Ivermectin 200 mcg/Kg/day and Albendazole 400 mg/day for 6 days	Not available	Alive
15	Hyperthyroidism Polycystic liver disease	Digestive Skin General malaise No Eosinophilia	May 2010: *S*.*stercoralis* larvae in fresh fecal samples and digestive fluids Serology +	70 days	Ivermectin 200 mcg/Kg/day and Albendazole 400 mg/day unknown duration	Not available	Alive
16	HIV infection *Cryptosporidium spp*. infection	Digestive Skin Renal General malaise Eosinophilia after ART was started	Mars 2006: *S*.*stercoralis* larvae in fresh fecal samples Serology +	34 days	Albendazole 400 mg/day for 5 days followed by Ivermectin 200 mcg/Kg/day for 2 days	Metronidazole 500 mg/8 hours Co-Trimoxazole 800/160 mg/12 hours Paromomycin 500 mg/6 hours Parenteral nutrition Fluid support	Alive
17	Leprosy Type 2 leprosy reaction	Digestive Eosinophilia after antiparasitic treatment was completed and steroids were discontinued	February 2010: *S*.*stercoralis* larvae in fresh fecal samples, and digestive biopsies. Serology not requested	15 days	Ivermectin 200 mcg/Kg/day for 2 days (again in 2 weeks)	Thalidomide 200 mg/day Dapsone 100 mg/day Clofazimine 50 mg/day Rifampicin 600 mg/month Prednisone 15 mg/day	Alive
18	Myelodysplastic syndrome with pancytopenia	Digestive General malaise No Eosinophilia	April 2015: *S*.*stercoralis* larvae in fecal samples, and duodenal and colonic biopsies. Serology +	30 days, in ICU	Ivermectin 200 mcg/Kg/day for 2 days	Omeprazole 80 mg/day Parenteral nutrition	Dead

HTLV: Human T-cell Lymphotropic Virus, HIV: Human Immunodeficiency Virus, HBV: Hepatitis B Virus, COPD: Chronic Obstructive Pulmonary Disease, ICU: Intensive Care Unit. ART: Antiretroviral Treatment.

Immunosuppressive conditions were identified in fourteen (77%) patients and were mainly due to prolonged steroids use (n = 9, 50%), followed by retroviral coinfections (four HIV, two HTLV). All the HIV cases were severely immunosuppressed, with CD4 counts of 8, 179, 10 and 22 at diagnosis (cases 5, 8, 9 and 16, respectively), and with detectable viral loads ranging from 390,000 to 4.300,000 copies/mL. In case 16, *S*.*stercoralis* severe infection was diagnosed along with HIV infection, while the others developed *S*.*stercoralis* hyperinfection syndrome within three months of starting antiretroviral therapy (ART). Of note, cases 5 and 9 had also received high dose steroids for cerebral toxoplasmosis before the onset of severe strongyloidiasis symptoms. Transplant preceded the clinical presentation in three patients, case 3 having received an allogenic blood stem cell transfer, and cases 7 and 13 having received a renal transplant. Case 2 developed hyperinfection syndrome after a renal transplant. Every transplanted case was also receiving steroids. A further two patients were under treatment with steroids; one for Sjögren syndrome, and the other for a type-2 lepromatous reaction (cases 11 and 17 respectively).

Other comorbidities were coinfection with *Trypanosoma cruzi* in three cases, hepatitis B virus in two cases, and *Mycobacterium leprae* and *Aspergillus spp*. in one case each.

All presented with digestive disorders. Ten (55%) patients presented with malaise. Eight patients (44%) had eosinophilia, as defined above, when strongyloidiasis was diagnosed; out of these, five were considered immunosuppressed. Of note, two out of the four HIV patients presented with eosinophilia, and case 16 (who was ART naïve at diagnosis) had an increase in his absolute and relative eosinophilia after starting ART, as happened with case 17 after steroid therapy was discontinued; Eosinophil count ranged from 191 eosinophils/μL (5.3%) to 11.200 eosinophils/μL (65.5%). Eight (44.4%) cases had fever, five (27.8%) had skin complaints, four (22.2%) presented with respiratory complaints and three (16.7%) with neurological disorders, one patient presented anemia, and one presented nephrotic syndrome.

Diagnosis was confirmed by identification of larvae in every case. Nine out of ten participating centers used Ritchie’s concentration technique and one obtained larvae after concentration with ethyl acetate. Larvae were cultured with charcoal culture, Harada-Mori filter paper or blood agar plate. Every case had a large number of filariform larvae in fresh stool samples (n = 16; 88.9%) concentration techniques (n = 6; 33.3%) or larval culture (n = 5; 27%). In some patients, *S*.*stercoralis* adult worms, eggs and larvae were identified in digestive and extra-digestive biopsies (n = 8; 44%), often described with an eosinophilic infiltrate around them. *S*.*stercoralis* forms were identified during necropsy in one case. In addition, ten (55%) had a positive serology, not performed in the remaining eight patients as the parasite had already been identified otherwise.

All the cases were treated with ivermectin adjusted to 200 mcg per kg a day, with the duration of treatment ranging from two to ten days. Three patients receiving ivermectin for two days had this regimen repeated one or two weeks later. Furthermore, six (33.3%) also received albendazole 400 mg before or concomitantly with ivermectin. One patient was treated with thiabendazole 1750 mg for three days in addition to a single dose of ivermectin. All needed inpatient management with a mean hospitalization stay of 25 days (range 1–164).Two cases (11.1%) needed intensive care and eventually died.

## Discussion

We found eighteen cases of severe *S*.*stercoralis* infection during the 15-year period studied in four different regions of Spain, most of which were imported, mainly from Latin America. The male to female ratio was 2:1, and the majority of patients were young adults. Immunosuppression frequently preceded the onset of symptoms, and mortality was lower than hitherto described, with a rate of 11.1%.

Robson et al. sought cases of *S*.*stercoralis* hyperinfection syndrome diagnosed in the United Kingdom after the Second World War to our date and described the largest series outside endemic areas [13]. Other recent studies on this topic are case reports, or hospital case-series describing any type of strongyloidiasis regardless of its severity [6, 14–16]. So ours is the biggest case series compilation outside endemic areas after that from Robson et al., who described severe cases over six decades.

One of the strengths of this study is the circumstances of the patients; since they were attended in reference centers, we have been able to fully describe the clinical picture and outcome of severe cases when all health-care resources are available in one place. However, this is also a disadvantage, as our data are not translatable to other clinical settings, nor to endemic areas. A further limitation of our study stems from the use of clinical records, as at times information was lacking due to the fact that it relied on the health worker’s thoroughness in registering data. For this reason, detailed information on exposure to risk factors was not available.

The age, sex and origin of the participants match the epidemiology described in previous hospital series in our country [6, 14–16], probably reflecting the epidemiology among our immigrant population. Clinical presentations are in accordance with those seen in previous case reports of severe strongyloidiasis, with a predominance of digestive tract disorders and general physical malaise. Nevertheless, these clinical symptoms might be undistinguishable from a complicated chronic *S*.*stercoralis* infection without microbiological evidence of a high number of larvae. Eosinophilia was a relatively frequent finding (n = 8, 44%) for such a population, since it is less frequently observed when cellular immunity is compromised. Of note, two of our patients with HIV developed eosinophilia once they started ART, and another presented eosinophilic infiltrate in a duodenal biopsy, findings which are in accordance with those described in other series [15, 17].

Regarding comorbidities, a recent systematic revision of severe strongyloidiasis cases described a large number as having received steroids before the onset of symptoms (67%), a 15% HIV coinfection rate, and a further 11.5% of cases being transplant recipients [9]. In our series, immunosuppression could be identified in two-thirds of the cases, predominantly among those receiving steroids and oncological chemotherapy, but four patients seemed to have no factor which would have triggered a massive increase in *S*.*stercoralis* larvae.

Serology is a sensitive tool although not highly specific. However, sensitivity can decrease to 42.9% in immnunocompromissed patients [18]. This technique was available in every center of our group. Since all our cases were confirmed with microbiological evidence, serology was only requested in ten of them. Out of these, six presented an immunosuppressive condition, raising the highest sensitivity described in such cases (100%), but we believe these numbers are not significant. Serology has also been recently proposed as a tool to monitor treatment success [16]. Unfortunately, it was not systematically requested during our patients’ follow-up.

Recent research into screening strategies for imported diseases found a significant rate of intestinal parasites among those awaiting treatment for oncohematological malignancies [19]. Steroid treatment and immunosuppressive drugs provided prior to and after transplants were both attributable causes of severe strongyloidiasis in half of the patients in our series, since *S*.*stercoralis* infection was thought to have been present before these therapies were started. Nevertheless, other recent reports have also described *S*.*stercoralis* infection in donors for both solid organ and stem cell transplants [20, 21]. Since the origin of the donors is unknown for the four patients who had received a transplant, they cannot be ruled out as a possible source of infection.

Some expert groups in tropical diseases have found rates of up to 18.4% of *S*.*stercoralis* infection in HIV patients coming from endemic areas, suggesting that this coinfection may be common [22]. Among those infected with HIV in our series, the development of severe strongyloidiasis could be attributed to HIV infection alone in only one patient (case 16), while a further three might have developed *S*.*stercoralis* severe infection as a manifestation of an immune reconstitution phenomenon, as described in the literature [23–25]. However, cases 5 and 9 were also receiving high-dose steroids as part of the treatment for a cerebral toxoplasmosis, which probably played a role in triggering a higher larval load.

An evidence-based guideline has been published during the development of this work. It recommends combination therapy with ivermectin and albendazole as the treatment of choice for severe cases [26], which was the empirical treatment given to 33% of our cases. The different treatment strategies used during our study period were chosen according to the published evidence at the time and drug availability, meaning ivermectin was not always present at the center when the patient arrived and albendazole or thiabendazole had to be launched initially, while awaiting ivermectin. Nevertheless, some drug regimens had to be repeated weeks apart, in some cases lasting up to months, since no parasitological and/or clinical improvement was observed with single dosages, probably reflecting prepatent autoinfections arising due to the increased larvae replication.

Buonfrate et al. found that all the patients suffering from severe strongyloidiasis who received ivermectin survived [9]. This treatment is the current drug of choice for the treatment of *S*.*stercoralis* infection and was given to every patient in our series [27, 28], what might explain the relatively low mortality described here (11.1%). In addition, they were managed in reference centers where the disease was suspected and diagnosed early, and where the coverage of concomitant sepsis with broad-spectrum antibiotics or intensive care were assured.

Raising awareness about the disease among populations-at-risk and healthcare professionals is strongly recommended [26]. During a *S*. *stercoralis* and *Trypanosoma cruzi* screening campaign, performed in 2016 among Latin-American immigrants in Alicante, Spain, it was found that 92.2% of participants (119/129) had never heard of strongyloidiasis, including none of the ten participants who had a positive *S*. *stercoralis* serology (personal communication). Moreover, a questionnaire about five Neglected Tropical Diseases completed by students from Madrid in their final year of Medicine, revealed that less than 18% of the students (18/103) ‘passed the exam’ on strongyloidiasis, this being one of the most worrying results [29].

We conclude that screening for strongyloidiasis should be mandatory for HIV patients, as well as for both transplant recipients and donors coming from endemic areas. Infection should also be ruled out in those diagnosed with HTLV-1 infection, and ideally before the onset of steroid treatment. At the time this paper was under revision, a panel of experts published an evidence-based guideline supporting this recommendation with a Ia grade [30]. It is clear that disease outcomes improve when clinicians are aware of the infection and ivermectin supply is available for patients who require it.
